# Diagnostic accuracy of low dose CT-Scan abdomen in patients with clinical features of acute appendicitis

**DOI:** 10.12669/pjms.40.9.9862

**Published:** 2024-10

**Authors:** Syed Jehanzeb Asim, Zubia Masood, Erum Soomro, Abdul Sami Qureshi

**Affiliations:** 1 Syed Jehanzeb Asim, Department of Emergency Medicine, Baqai Medical University, Karachi, Pakistan; 2 Dr. Zubia Masood, Department of General Surgery, Baqai Medical University, Karachi, Pakistan; 3 Dr. Erum Soomro, Department of Emergency Medicine, Ziauddin University, Karachi, Pakistan; 4 Dr. Abdul Sami Qureshi, Department of Emergency Medicine, Ziauddin University, Karachi, Pakistan

**Keywords:** Acute appendicitis, Low Dose CT scan, Diagnostic Accuracy

## Abstract

**Background & Objective:**

Acute appendicitis is one of the commonest causes of acute abdominal pain presenting to emergency department (ED) and Computerized Tomography scan (CT) is considered gold standard for its diagnosis. Internationally Low Dose Computerized Tomography scan (LDCT) in emergency department is recommended as a beneficial tool to diagnose acute appendicitis with less exposure to radiation and reduction in the rate of negative laparotomy. Local trials are needed to determine the diagnostic accuracy of LDCT as the first line imaging test for acute appendicitis. Our objective was to determine the diagnostic accuracy of LDCT as the first line imaging test for acute appendicitis.

**Methods:**

An observational study was conducted over a sample of 147 patients presented with suspected acute appendicitis to the emergency department of Ziauddin University Hospital, Karachi from November 2018 till May 2019. Non–probability consecutive technique used. Aged ≥ 16 years presented in emergency department with the history (symptoms) and physical examination (Signs) suspecting acute appendicitis were included. Patients with contraindications to CT scan e.g. pregnant women. Patients with signs of Acute Peritonitis requiring immediate surgery. CT scan refused by the patient or patient’s attendant were excluded. Histopathology was the gold standard in diagnosing acute appendicitis. The data was analyzed using open epi sample size calculator.

**Results:**

One hundred forty six patients had positive findings on LDCT for acute appendicitis (99.3%) whereas only one patient had negative findings (0.7%). The sensitivity and specificity of LDCT for the detection of acute appendicitis were estimated as 96.45% and 16.67% by taking histopathology as gold standard. Negative predictive value (NPV) and positive predictive value (PPV) were estimated as 16.67% and 96.45% respectively. The overall accuracy of LDCT was 93.88%.

**Conclusion:**

Our study showed that for diagnosing acute appendicitis, LDCT is harmless, fast and economical imaging modality and has diagnostic accuracy with decrease in radiation dose.

## INTRODUCTION

In emergency department, patients with acute appendicitis frequently presents with a complain of acute abdomen, making it quite challenging for the emergency physicians to diagnose accurately. Acute appendicitis is frequently seen in younger patients and 5% to 10% in elderly patients.^1^ Treatment of choice for acute appendicitis is open or laparoscopic appendectomy. However, literature shows 15% to 39% of negative appendectomy.^2^

The diagnosis and management of acute Appendicitis is based mainly on symptoms and sign. Different tools like Alvarado scoring system also supports the diagnosis of acute appendicitis. An Alvarado Score for acute appendicitis showed 91.7% and 50% sensitivity and specificity, respectively. According to Alvarado Score, Appendicitis is divided into Low Risk, which is from 1-4, medium risk which is 5-6 and High Risk, which is from 7-10.^3^-^7^

In acute Appendicitis, sonographic evaluation is the first investigation for diagnosis with sensitivity and specificity of 77.2% and 60%, respectively.^8^,^9^ It has the advantages of being easily accessible, low-cost, and with no radiation hazards however its operator dependant.^10^ Negative appendectomy rate was found to be 3% to 15% after ultrasound abdomen.^7^,^11^

A meta-analysis supports that the use of preoperative abdominal CT is associated with lower negative appendectomy rate, literature also supports CT being gold standard radiology in diagnosing acute appendicitis.^4^,^10^

LDCT has an edge over the ultrasound for being more specific and has low incidence of negative appendectomy rate. LDCT is accurate and avoids late diagnosis. LDCT is cost effective than standard CT scan (SDCT) and also has less radiation exposure. For diagnosis of acute Appendicitis, LDCT had no suggestive analytical differentiation in the separate BMI subcategory. In patients with BMI from 18.5 till 25, LDCT is helpful in diagnosis.^10^ This study aimed to determine the diagnostic accuracy of LDCT abdomen in cases with suspected acute appendicitis.

## METHODS

An observational study was conducted on patients with the suspicion of acute appendicitis presented in the Emergency Department (ED), Ziauddin University Hospital, Karachi. The duration of the study lasted from November 2018 till May 2019. A total of 147 patients were recruited implying the non–probability consecutive sampling technique.

### Ethical Approval:

The ethical approval for this study was taken from ethical review committee (ERC) of Ziauddin University given the reference number: 0501018JAEMD, Dated: September 5, 2018.

The sample size was calculated using the open epi calculator, considering the expected sensitivity of 0.37 and specificity of 0.80 and achieving a prevalence of acute appendicitis was 0.14, confidence level of 95%.

Sample size n = [DEFF*Np(1-p)]/ [(d2/Z21- “α” /2*(N-1) +p*(1-p)]

### Inclusion Criteria:

- On the basis of history (symptoms) and physical examination (signs) and aging 16yrs and above were recruited.

### Exclusion Criteria:

- Contraindicated to CT scan e.g.: pregnant women.
- CT scan refused by the patient or patient’s attendant.

LDCT scan was performed on all the patients after taking written consent. Criteria for positive acute appendicitis in LDCT were wall thickening, strong mucosal enhancement, luminal distension more than 6mm, appendicolith and periappendiceal fat infiltration. Reporting of LDCT was done by radiologist. After the surgery, sample was sent to histopathology to rule out the presence of appendicitis. Histopathology was taken as a gold standard. Histopathology confirmed the diagnosis of AA which was then matched with the findings of LDCT.

Results were analyzed using SPSS version 20 to reach a conclusion. Quantitative variables include dependent finding of Low dose CT scan and Histopathology report. Sensitivity and specificity was calculated by using two by two tables.

## RESULTS

**Fig.1:**
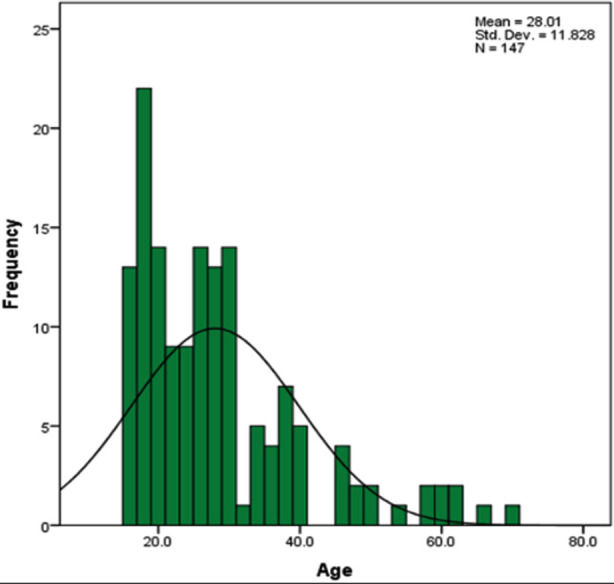
Descriptive statistics of age.

The mean age of the patients was estimated as 28 ± 11.92 years (Range: 16-70 years) (Fig.1). Majority of the patients were males 63.3% and females were 36.7%. Out of 147 Patients, 57 had positive findings whereas 90 had negative findings, on ultrasound. Out of 147 patients, 146 had positive findings on LDCT for acute appendicitis whereas only one patient had negative finding (Table-I). Out of 147 patients, 141 patients had positive findings and six patients had negative findings on histopathology for acute appendicitis (Table-II). Of 147 patients, most of the patients were of age less than 30 years 99 (67.3%) and 48 (32.7%) were of age equal to or more than 30 years.

**Table-I:** Findings on LDCT & Histopathology for Acute Appendicitis.

	LDCT	Histopathology	Percentage (LDCT / Histopathology)
Negative	1	6	0.7% / 4.1%
Positive	146	141	99.3% / 95.9%

Total	147	147	100%

**Table-II:** Diagnostic Accuracy of LDCT for Diagnosing Appendicular Inflammation by Histopathology as Gold Standard.

	Histopathology		

LDCT	Positive	Negative	Total
Positive	136	5	141
Negative	5	1	6

Total	141	6	147

Sensitivity= 96.45%, Specificity= 16.67%, Negative predictive value (NPV) = 16.67%, Positive predictive value (PPV) = 96.45%, Diagnostic accuracy (DA) = 93.19%.

The sensitivity and specificity of LDCT for the detection of appendicular inflammation were estimated as 96.45% and 16.67% by taking histopathology as a gold standard. Whereas, NPV and PPV were estimated as 16.67% and 96.45% respectively. The overall accuracy of LDCT was 93.88%.

## DISCUSSION

LDCT is beneficial in diagnosing acute appendicitis. Overall, LDCT is as good as standard CT scan. LDCT and SDCT accomplish the same functioning in diagnosing appendicular inflammation with BMI > 18.5. In our research, the mean age of the patients was estimated as 28.01±11.92 years (Range: 16-70 years). LDCT has a sensitivity and specificity of 100% and16.67%, respectively. PPV and NPV of LDCT are 96.58% and 100%, respectively. Overall, the accuracy of LDCT scan was 96.6%. LDCT has a low tube current-time product which is 30mAs, pitch 10mm, gantry rotation time 0.5 second and a volume dose index of 2.1mGy. SDCT has a low tube current –time product which is 180mAs, pitch 5mm, gantry rotation time 1.0 second and a volume index of 12.6mGy.

In a study conducted in Pakistan, 185 patients (101 males and 84 females) were included. Age group between 18 to 60 years (mean age: 33.36 ± 10.35 years). Sensitivity and Specificity of LDCT were 94.64% and 93.15%, respectively. Radiation dose of LDCT was 2mSv. Results show that LDCT should be used for the diagnosis of acute appendicitis to avoid excessive radiations exposure. The results of our study match with results of that study too.^1^

In another study conducted in India, 83 patients (44 males and 39 females) were included. Mean age was 37 years (Range: 18 to 85 years). Range of BMI was 16.44 – 31.53 kg/m^2^ (mean: 22.97 kg/m2 ± 3.06). LDCT and SDCT, tube current time product was 30mA and 250mA, respectively and mean effective radiation dose was 1.47 mSv ± 0.9 and 12.30 mSv ± 0.82, respectively. For LDCT, Sensitivity and Specificity was 98.2% and 100%, respectively. Result showed that LDCT was very much accurate in diagnosing acute appendicitis which are similar to our results.^10^

In yet another study conducted in South Korea, 891 patients were included. In suspected of acute appendicitis, 444 patients had LDCT and 447 patients had SDCT. In LDCT and SDCT, negative appendectomy rate was 3.5% and 3.2%, respectively. Result shows with respect to the negative appendectomy, LDCT and SDCT had similar effect in diagnosing inflamed appendix. They concluded that role of LDCT should be the first technique in diagnosing appendicular inflammation.^11^

In a study conducted in Finland. 856 patients (411 were male and 445 were females). Median age was 37 years (age range: 16-87years). LDCT and SDCT had median radiation dose was 3mSv and 7 mSv, respectively. Sensitivity and specificity of LDCT was 90% and 91%, respectively. BMI was less than (<) 30kg/m². Result showed LDCT and SDCT had similar performance in diagnosing inflamed appendix. In the diagnosing appendicular inflammation, sensitivity and specificity of LDCT and SDCT were unchanged, supporting our study results.^12^

In a similar study conducted in republic of Korea, 102 patients (47 men and 55 women) were included. Age group between 15 to 82 years (Mean age: 41.2 years). The mean BMI was 21.8 ± 4.6 kg/m^2^. Tube current time product of LDCT was 30mAs and 100mAs. Result shows LDCT_30mAs_ and LDCT_100mAs_ have similar diagnostic accuracy in the diagnosing acute appendicitis.^13^

In a study conducted in Canada, 531 patients were included. Patients were divided into two groups: First, Low Dose CT scan and Second, Standard Dose CT scan. In Low Dose CT scan, 181 patients (70 men and 111 females) were included. The mean age was 26 ± 6 years (Range: 17 to 53 years). In Standard CT scan, 350 patients (Male: 146 men and 206 females) were included. The mean age was 55 ± 17 years (Range: 19 to 93 years). Result shows LDCT and SDCT had same diagnostic accuracy in the diagnosing appendicular inflammation and LDCT should be the first imaging modality in the diagnosing appendicular inflammation.^14^ The results of our study match with results of this study too.

In another study conducted in Taiwan, 101 patients (44 males and 57 females) were included. Age group between 21 to 71 years (mean age: 38.9 ± 12.6 years). The mean BMI was 23.3 ± 3.6. LDCT had tube current time product was 49mAs. Result shows that LDCT scans were studied by junior radiologist or by senior radiologist. Senior radiologist gave effective results in diagnosing acute appendicitis on LDCT than junior radiologist. In this study patients were managed conservatively, the final diagnoses were based on discharge diagnosis and clinical follow-up and no histopathology was done.^15^ The results of this study are inconsistent with our study. The final diagnoses in our study are based on LDCT, whether managed conservatively or surgically.

In a study conducted in Korea, 257 patients (111 were male and 146 were female) were included. Mean age was 26.6 years ± 7.5 (range: 15 to 40 years). LDCT and SDCT, effective tube current time product ranged between 25 to 40 mAs and 110 to 200 mAs, respectively. Result shows LDCT and SDCT had same diagnostic accuracy in the diagnosing appendicular inflammation and LDCT should be the first imaging modality in the diagnosing appendicular inflammation.^16^ The results of our study match with results of this study too.

In yet another study conducted in Korea, 2957 patients were included. Two groups were included. First, Low Dose CT scan (LDCT) and Second, Standard Dose CT scan (SDCT). In LDCT and SDCT, 1766 and 1680 patients were included, respectively. In LDCT, 781 were male and 985 were female. In SDCT, 762 were male and 918 were female. Effective radiation dose of LDCT was less than 2 mSv (Range: 1.5 to 4.2 mSv). Result showed that LDCT was very much accurate in diagnosing acute appendicitis and LDCT should be the first imaging modality in the diagnosing appendicular inflammation.^16^ The results of our study match with results of that study too.

### Limitations:

An identifiable limitation of our study is that the findings of LDCT were not compared with standard CT scan. Moreover, the research was conducted only in one hospital setting.

### Strengths of the study:

The strength of our study is a good sample size and the point that all cases diagnosed as appendicitis on LDCT were confirmed by histopathology.

## CONCLUSION

In diagnosis of acute appendicitis, LDCT has increased diagnostic accuracy with decrease in radiation dose. LDCT is safe, fast and economical imaging modality in patients with right lower quadrant pain.

### Author’s contribution:

**SJA** conceptualized the study, collected the data and prepared the manuscript.

**ZM** overall supervision and helped in preparation of the manuscript.

**ES** helped in collecting the data.

**ASQ** helped in literature search and data analysis.

All authors reviewed the paper, gave final approval, and agreed to be accountable for all aspects of the work.

## References

[1] Ali R, Raja R, Arooj S (2019). Diagnostic Accuracy of Low Radiation Dose Contrast Enhanced Abdominal CT in Acute Appendicitis. Pak J Med Res.

[2] Alia P, Afsounb P, Alirezaa G (2019). Appendicitis:Clinical implications in negative appendectomy. IJS Open.

[3] Pifeleti S, Hansell D, Kaspar A (2022). Sensitivity and specificity of the Alvarado Score for the timely differential diagnosis of acute appendicitis for a case series in Samoa. Ann Med Surg (Lond).

[4] Krajewski S, Brown J, Phang PT, Raval M, Brown CJ (2011). Impact of computed tomography of the abdomen on clinical outcomes in patients with acute right lower quadrant pain:a meta-analysis. Can J Surg.

[5] Teng TZJ, Thong XR, Lau KY, Balasubramaniam S, Shelat VG (2021). Acute appendicitis-advances and controversies. World J Gastrointest Surg.

[6] Bouali M, El Berni Y, Moufakkir A, El Bakouri A, El Hattabi K, Bensardi F (2022). Value of Alvarado scoring system in diagnosis of acute appendicitis. Ann Med Surg (Lond).

[7] Fu J, Zhou X, Chen L, Lu S (2021). Abdominal Ultrasound and Its Diagnostic Accuracy in Diagnosing Acute Appendicitis:A Meta-Analysis. Front Surg.

[8] Aly NE, McAteer D, Aly EH (2016). Low vs. standard dose computed tomography in suspected acute appendicitis:Is it time for a change?. Int J Surg.

[9] Chaochankit W, Boocha A, Samphao S (2022). Negative appendectomy rate in patients diagnosed with acute appendicitis. BMC Surg.

[10] Singh NR, Luwang NT, Priyabarta Y, Singh CG, Singh WJ (2018). Low-dose noncontrast computed tomography in adults with acute appendicitis. J Med Soc.

[11] Kim K, Kim YH, Kim SY, Kim S, Lee YJ, Kim KP (2012). Low-dose abdominal CT for evaluating suspected appendicitis. N Engl J Med.

[12] Haijanen J, Sippola S, Tammilehto V, Grönroos J, Mäntyoja S, Löyttyniemi E (2021). Diagnostic accuracy using low-dose versus standard radiation dose CT in suspected acute appendicitis:prospective cohort study. Br J Surg.

[13] Kim SH, Yoon JH, Lee JH, Lim YJ, Kim OH, Ryu JH (2015). Low-dose CT for patients with clinically suspected acute appendicitis:optimal strength of sinogram affirmed iterative reconstruction for image quality and diagnostic performance. Acta Radiol.

[14] Dowhanik A, Tonkopi E, Crocker CE, Costa AF (2021). Diagnostic performance and radiation dose of reduced vs. standard scan range abdominopelvic CT for evaluation of appendicitis. Eur Radiol.

[15] Chang CC, Wong YC, Wu CH, Chen HW, Wang LJ, Lee YH (2016). Diagnostic Performance on Low Dose Computed Tomography for Acute Appendicitis Among Attending and Resident Radiologists. Iran J Radiol.

[16] Yun SJ, Ryu CW, Choi NY, Kim HC, Oh JY, Yang DM (2017). Comparison of Low- and Standard-Dose CT for the Diagnosis of Acute Appendicitis:A Meta-Analysis. Am J Roentgenol.

